# Magnetic resonance imaging and molecular features associated with tumor-infiltrating lymphocytes in breast cancer

**DOI:** 10.1186/s13058-018-1039-2

**Published:** 2018-09-03

**Authors:** Jia Wu, Xuejie Li, Xiaodong Teng, Daniel L. Rubin, Sandy Napel, Bruce L. Daniel, Ruijiang Li

**Affiliations:** 1 Department of Radiation Oncology, Stanford University School of Medicine, 1070 Arastradero Road, Stanford, CA 94305 USA; 2 Department of Pathology, First Affiliated Hospital of Zhejiang University, Hangzhou, 310058 Zhejiang China; 3 Department of Radiology, Stanford University School of Medicine, Stanford, CA 94305 USA; 4 Department of Biomedical Data Science, Stanford University School of Medicine, Stanford, CA 94305 USA; 5 Center for Biomedical Informatics Research, Department of Medicine, Stanford University School of Medicine, Stanford, CA 94305 USA

**Keywords:** Tumor-infiltrating lymphocytes, Imaging marker, Cytolytic score, Nonsynonymous mutation burden, Breast cancer

## Abstract

**Background:**

We sought to investigate associations between dynamic contrast-enhanced (DCE) magnetic resonance imaging (MRI) features and tumor-infiltrating lymphocytes (TILs) in breast cancer, as well as to study if MRI features are complementary to molecular markers of TILs.

**Methods:**

In this retrospective study, we extracted 17 computational DCE-MRI features to characterize tumor and parenchyma in The Cancer Genome Atlas cohort (*n* = 126). The percentage of stromal TILs was evaluated on H&E-stained histological whole-tumor sections. We first evaluated associations between individual imaging features and TILs. Multiple-hypothesis testing was corrected by the Benjamini-Hochberg method using false discovery rate (FDR). Second, we implemented LASSO (least absolute shrinkage and selection operator) and linear regression nested with tenfold cross-validation to develop an imaging signature for TILs. Next, we built a composite prediction model for TILs by combining imaging signature with molecular features. Finally, we tested the prognostic significance of the TIL model in an independent cohort (I-SPY 1; *n* = 106).

**Results:**

Four imaging features were significantly associated with TILs (*P* < 0.05 and FDR < 0.2), including tumor volume, cluster shade of signal enhancement ratio (SER), mean SER of tumor-surrounding background parenchymal enhancement (BPE), and proportion of BPE. Among molecular and clinicopathological factors, only cytolytic score was correlated with TILs (ρ = 0.51; 95% CI, 0.36–0.63; *P* = 1.6E-9). An imaging signature that linearly combines five features showed correlation with TILs (ρ = 0.40; 95% CI, 0.24–0.54; *P* = 4.2E-6). A composite model combining the imaging signature and cytolytic score improved correlation with TILs (ρ = 0.62; 95% CI, 0.50–0.72; *P* = 9.7E-15). The composite model successfully distinguished low vs high, intermediate vs high, and low vs intermediate TIL groups, with AUCs of 0.94, 0.76, and 0.79, respectively. During validation (I-SPY 1), the predicted TILs from the imaging signature separated patients into two groups with distinct recurrence-free survival (RFS), with log-rank *P* = 0.042 among triple-negative breast cancer (TNBC). The composite model further improved stratification of patients with distinct RFS (log-rank *P* = 0.0008), where TNBC with no/minimal TILs had a worse prognosis.

**Conclusions:**

Specific MRI features of tumor and parenchyma are associated with TILs in breast cancer, and imaging may play an important role in the evaluation of TILs by providing key complementary information in equivocal cases or situations that are prone to sampling bias.

**Electronic supplementary material:**

The online version of this article (10.1186/s13058-018-1039-2) contains supplementary material, which is available to authorized users.

## Background

Immunotherapy for treating patients with cancer has generated much excitement in recent years [1]. Compared with conventional therapies, immune checkpoint blockade (ICB) such as anti-PD1 therapy has achieved durable clinical response and long-term survival benefit in a variety of cancer types [2, 3]. However, only a small proportion of patients respond to current immunotherapy, underscoring the need for predictive biomarkers to identify appropriate patients [4]. One promising biomarker is tumor-infiltrating lymphocytes (TILs), because it is now recognized that a preexisting antitumor immunity is required for the success of ICB-based immunotherapy [5]. In breast cancer, there is strong evidence for the prognostic and predictive value of TILs [6]. Several large clinical trials have demonstrated that TILs are associated with pathological complete response and prognosis after chemotherapy or targeted therapies, particularly in triple-negative breast cancer (TNBC) and human epidermal growth factor receptor 2 (HER2)-positive breast cancer [7–14].

The evaluation of TILs involves visualization and measurement of lymphocytes on H&E-stained histological slides of tumor samples [15]. Current guidelines issued by the International Immuno-Oncology Biomarker Working Group on Breast Cancer recommend that evaluation of TILs be performed in the stromal rather than intraepithelial compartments, and preferably on whole tumor sections over core biopsies [16]. Despite numerous efforts to standardize the evaluation of TILs, this process remains laborious and subjective with inter- and intrarater variability [16]. Moreover, evaluation of TILs in the preoperative neoadjuvant setting is problematic because of heterogeneous tumor shrinkage patterns and sampling bias in a biopsy. A more objective, consistent method to evaluate TILs in breast cancer would be extremely valuable.

Imaging allows noninvasive visualization of the entire tumor and its surrounding tissue. Recent studies have demonstrated associations between specific magnetic resonance imaging (MRI) features and pathological or molecular patterns, such as molecular subtypes [17–22] and gene expression signatures or pathways [23–28]. These data support the underlying pathophysiology of the disease being reflected on imaging at a macroscopic level, and this link may be revealed by a more detailed comprehensive image analysis.

The purpose of this study was to investigate the association between MRI features and TILs in breast cancer. We explored whether computational imaging features could be used to predict TILs. Further, we constructed a composite prediction model by integrating imaging and immune-related molecular features and validated its clinical relevance in an independent cohort.

## Methods

### Study design

**Fig. 1:**
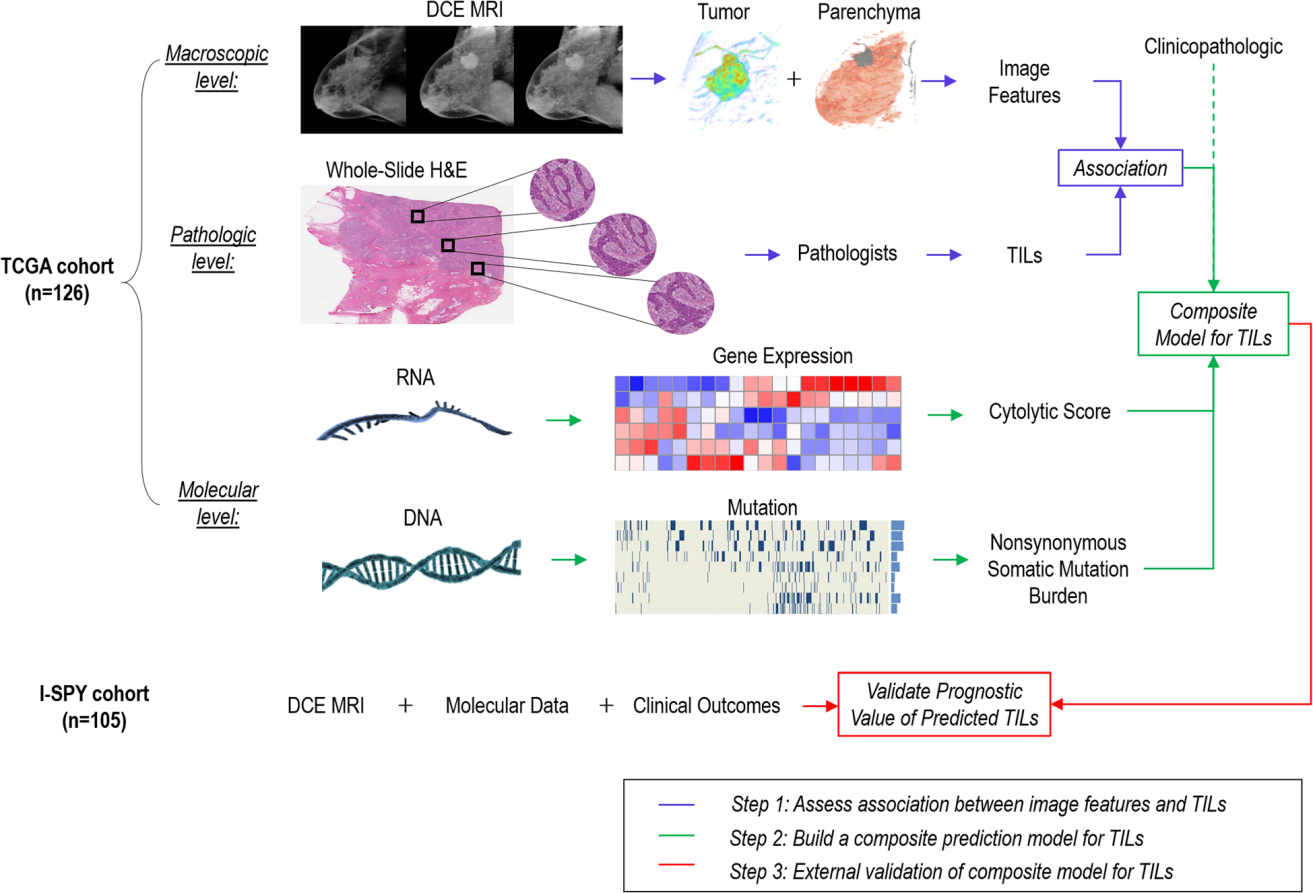
Flowchart of the study design, which included three main steps (color-coded). *DCE* Dynamic contrast-enhanced, *MRI* Magnetic resonance imaging, *TCGA* The Cancer Genome Atlas, *TILs* Tumor-infiltrating lymphocytes

We carried out this institutional review board-approved, Health Insurance Portability and Accountability Act (HIPAA)-compliant retrospective study in three steps (Fig. 1). First, we characterized both tumor and parenchymal enhancement patterns at dynamic contrast-enhanced (DCE) MRI and evaluated their association with TILs. Second, we built a composite model to predict TILs by integrating imaging with molecular and clinicopathological data. Third, we tested the prognostic significance of the TIL model in an independent cohort.

### Patient cohorts

We analyzed two breast cancer cohorts from The Cancer Genome Atlas (TCGA) project [29] and the I-SPY 1 (Investigation of Serial Studies to Predict Your Therapeutic Response with Imaging And moLecular Analysis) trial [30]. For this study, the inclusion criteria for TCGA cohort were (1) pathologically proven invasive carcinomas, (2) pretreatment DCE-MRI data available, (3) H&E-stained whole-tumor tissue sections available, and (4) tumor gene expression data from RNA-sequencing (RNA-seq) and mutational data from whole-exome sequencing available. We applied similar inclusion criteria to select patients from the I-SPY 1 cohort, except that outcomes were available, but H&E-stained slides and mutational data were not required. After selection, 126 patients from TCGA and 105 patients from I-SPY 1 were eligible for the proposed study. The detailed selection procedures are shown in Additional file 1: Figure S1. Clinical and imaging data are publicly available for both cohorts from The Cancer Imaging Archive (TCIA) (www.cancerimagingarchive.net).

### Evaluation of tumor-infiltrating lymphocytes

TILs were evaluated for TCGA cohort, for which detailed biospecimen collection and processing protocols have been described elsewhere [29]. In brief, the tumor sections were collected from surgical specimens and reviewed by a board-certified pathologist to confirm the presence of invasive carcinoma. The H&E-stained whole-slide tumor sections were digitally scanned and are available from the Cancer Digital Slide Archive (http://cancer.digitalslidearchive.net/).

Two pathologists (XT and XL, with 30 and 5 years of experience, respectively, in reading breast cancer tissue slides) evaluated TILs in consensus based on the recommendations from the International Immuno-Oncology Biomarker Working Group on Breast Cancer [16]. Two pathologists simultaneously reviewed the digital slides of each patient from the Cancer Digital Slide Archive, and the TILs were measured as the percentage of lymphocytes and macrophages in the area of total intratumoral stromal compartments. In addition, three discrete categories are defined, with ≤ 10%, > 10% to ≤40%, and > 40% to ≤ 90% TILs indicating tumors with no/minimal, intermediate, and high lymphocyte infiltration, respectively [16]. To assess interrater variability, we calculated the intraclass correlation coefficient (ICC) between our TIL percentage and those reported in a previous study focused on TNBC [31] for 15 overlapped cases in TCGA cohort.

### Imaging protocols

The detailed imaging protocol for TCGA cohort has been reported elsewhere [27]. In brief, the scans were performed between September 1999 and June 2006 at six participating centers with a 1.5-T or 3-T GE Healthcare (Milwaukee, MI, USA), Siemens (Erlangen, Germany), or Philips (Amsterdam, The Netherlands) whole-body MRI system with a standard double-breast coil. The dynamic protocol of DCE-MRI was in accordance with the American College of Radiology guidelines, which included one precontrast and two to seven postcontrast scans (with a gadolinium-based contrast agent), in either the sagittal or axial view.

The detailed imaging protocol for the I-SPY cohort was reported elsewhere [32, 33]. To match the MRI from TCGA cohort, we focused on the scans acquired before neoadjuvant chemotherapy (i.e., baseline scans). MRI was performed through a 1.5-T GE Healthcare, Siemens, or Philips system, with a dedicated breast radiofrequency coil. The DCE-MRI protocols include one precontrast scan and two postcontrast phases with one ~ 2.5 minutes and another one ~ 7.5 minutes.

### Image processing and harmonization

Given the diverse imaging protocols within the multicenter TCGA data and I-SPY 1 cohorts, we developed a pipeline to normalize the imaging data before extracting quantitative feature. First, we applied the N4 bias correction to correct for shading artifacts. Next, we standardized the temporal resolution of DCE-MRI scans in TCGA and I-SPY cohorts. In particular, for each patient, we included DCE-MRI before contrast agent administration and two postcontrast scans, with one having a 2–3-minute delay and the other having an ~ 7.5-minute delay. Third, to explicitly account for heterogeneous imaging protocols, for each individual, the voxel values of DCE-MRI were normalized by the parenchyma without contrast (i.e., the average value of interquartile voxel from parenchyma before administrating contrast). Finally, the MRI scans were resized to have an isotropic voxel resolution of 1 mm to assure consistent and meaningful computation of 3D textural features.

### Tumor and background parenchyma segmentation

The detailed process used for segmentation was reported elsewhere [28, 34]. Briefly, two radiologists with 14 and 11 years of experience, respectively, in breast imaging manually delineated the 3D tumor slice-by-slice and reached consensus regarding 3D tumor contours. The ipsilateral parenchyma was segmented automatically through Fuzzy C-means clustering. The 3D parenchymal segmentation was inspected by two radiologists, and they manually revised it when necessary.

### MRI feature exaction

The rationale of feature extraction is to provide a comprehensive characterization of breast cancer at DCE-MRI. We initially extracted 110 computational imaging features as defined in a previous study [28] and removed those with linear ICCs above 0.85. For correlated features, the one that showed highest robustness with respect to tumor contour variations (manual segmentation vs automatic segmentation via Fuzzy C-means clustering) was kept, similar to previous studies [34, 35]. As a result, 17 nonredundant imaging features remained. The selected features characterize the tumoral and parenchymal phenotypes at DCE-MRI, which include five tumor morphological features, four tumor texture features, two functional tumor volume features, four background parenchymal enhancement (BPE) features, and two tumor-surrounding PE features. The mathematical formulation and the interpretation and clinical relevance [27, 28, 32, 36–38] of these features are elaborated in Table 1. The computation of all imaging features was implemented automatically with MATLAB software (MathWorks, Natick, MA, USA).

**Table 1:** Definition and interpretation of 17 computational imaging features extracted from dynamic contrast-enhanced magnetic resonance imaging scans

Type	No.	Definition	Interpretation
Morphology (M)	5	M1: Volume	Tumor shape, size, and boundary smoothness (i.e., descriptors according to BI-RADS classification)
		M2: Sphericity	
		M3: Surface-to-volume ratio	
		M4: Mean of margin sharpness	
		M5: SD of margin sharpness	
Texture of kinetic maps (TEX)	4	TEX1: Correlation of SER	Spatial tumor heterogeneity of the SER map
		TEX2: Cluster shade of SER	
		TEX3: Energy of SER	
		TEX4: Entropy of SER	
Functional tumor volume (FTV)	2	FTV1: Absolute volume of the active tumor with SER > 1.0	Subvolume of tumor with fast contrast uptake and washout
		FTV2: Absolute volume of the active tumor with SER > 1.5	
Ipsilateral background parenchymal enhancement (BPE)	4	BPE1: Absolute volume of BPE with PE > 0.2	Enhanced subvolume of ipsilateral breast parenchyma at the early postcontrast phase, in accordance with the BI-RADS classification
		BPE2: Absolute volume of BPE with PE > 0.6	
		BPE3: Relative volume of BPE with PE > 0.2	
		BPE4: Relative volume of BPE with PE > 0.6	
Tumor surrounding background parenchymal enhancement (TS-BPE)	2	TS-BPE1: Mean value of PE in tumor surrounding parenchyma (2 cm)	Enhancement of parenchyma surrounding the tumor within 2-cm distance
		TS-BPE2: Mean value of SER in tumor surrounding parenchyma (2 cm)	

*BI-RADS* Breast Imaging Reporting and Data System, *PE* percent enhancement; $PE=\frac{I_{early\ postcontrast}-{I}_{precontrast}}{I_{precontrast}}$, *SER* signal enhancement ratio; $SER=\frac{I_{early\ postcontrast}-{I}_{precontrast}}{I_{late\ postcontrast}-{I}_{precontrast}}$

### Molecular features related to tumor-infiltrating lymphocytes

Tumor mutation burden is an important genetic factor in mediating antitumor immunity. Tumors with a higher mutation load are associated with a higher neoantigen level and thus are more immunogenic and likely to have higher immune infiltration and more TILs [39]. The cytolytic activity reflects local immune effector function and can indicate the presence of TILs. Indeed, cytolytic activity computed from the gene transcript levels of two critical immune cytolytic effectors [40], perforin (*PRF1*) and granzyme A (*GZMA*), has been shown to be closely related to immune infiltration and CD8+ T-cell activation [41, 42]. For TCGA breast cancer cohort, gene expression data from RNA-seq and mutational data from whole-exome sequencing are available in the Genomic Data Commons (https://gdc.cancer.gov/). On the basis of these data, we computed the nonsynonymous somatic mutational burden and cytolytic activity score, defined as the geometric mean of the expression of two genes: *GZMA* and *PRF1* [40]. Similarly for the I-SPY 1 cohort, we computed the cytolytic activity score on the basis of microarray gene expression data available from the Gene Expression Omnibus (https://www.ncbi.nlm.nih.gov/geo/; [GEO:GSE22226]) [43]. The ComBat algorithm [44] was implemented to harmonize the gene expression data from TCGA and I-SPY.

### Association with tumor-infiltrating lymphocytes and predictive modeling

We first evaluated the Pearson linear correlation between individual imaging features and percentage of TILs in TCGA cohort. Next, we built a predictive model for TILs by combining multiple imaging features into an imaging signature. For this purpose, we used linear regression with feature selection via LASSO (least absolute shrinkage and selection operator) [45] to avoid overfitting. In addition, tenfold cross-validation was applied and repeated 100 times to minimize the selection bias. The most frequently selected imaging features (> 90%) were used to fit the final model. Further, we investigated whether combining the imaging signature with immune-related molecular features (cytolytic score and somatic mutation burden) would improve prediction accuracy for TILs by fitting a composite model via multivariate linear regression.

### Performance evaluation

To evaluate the prediction models, we calculated the Pearson linear correlation between pathologist-rated and estimated percentage of TILs. In addition, patients were divided into three recognized TIL categories (low, intermediate, and high immune infiltration) [16], and pairwise classification among the three categories was evaluated. We compared the performance of the composite model with molecular features based on cytolytic score and imaging signature. In particular, the ROC analysis and AUC were used to assess the binary prediction accuracy of the models. The threshold used to separate different prediction models was defined on the basis of Youden’s J statistics [46], and the corresponding sensitivity, specificity, and accuracy were reported. Finally, we tested prognostic significance of the imaging signature as well as the composite TIL model by assessing their association with recurrence-free survival (RFS) in the entire I-SPY 1 cohort as well as in clinically relevant subgroups according to the receptor status. Because the prognostic value of TILs seems to be strongest in TNBC [11, 13], we expect that the composite model would also be prognostic within the TNBC subgroup in the I-SPY 1 cohort.

### Statistical analysis

In univariate analysis, to adjust for multiple statistical testing, the Benjamini-Hochberg method was used to control the false discovery rate (FDR). The Mann-Whitney *U* statistic was used to assess the statistical significance of binary classification of TIL categories by comparing the prediction models with a random guess with an AUC of 0.5. The DeLong test was used to determine the 95% CIs and compute *P* values for the comparison of ROC curves. The Cox proportional hazards model was used to build survival models. Kaplan-Meier analysis was used to estimate survival probability. The log-rank test and concordance index were used to assess prognostic performance. All statistical tests were two-sided. *P* value < 0.05 and FDR < 0.2 were considered to be statistically significant. Statistical analysis was performed in R (R Foundation for Statistical Computing, Vienna, Austria).

## Results

### Patient characteristics and tumor-infiltrating lymphocyte evaluation

Among 1098 cases in TCGA breast cancer cohort, 126 patients were eligible for our study. A majority (*n* = 92, 73%) of patients had low immune infiltration (0–10% TILs) in their tumor stroma, whereas 20% (*n* = 25) and 7% (n = 9) of patients had intermediate and high immune infiltration, respectively. Clinicopathological characteristics of patients in each of the three TIL categories are shown in Table 2. There was high reproducibility between TILs measured by our pathologists and previously reported values with ICC of 0.80 (*P* = 0.002). For the I-SPY 1 cohort, 105 patients were eligible and included in this study (patient characteristics summarized in Additional file 2: Table S1).

**Table 2:** Clinical and pathological Characteristics for Eligible Patients in the TCGA Cohort

Parameter	≤ 10% stromal TILs	> 10 to ≤ 40% stromal TILs	> 40 to ≤ 90% stromal TILs	*P* value^a^
	Tumor with no/minimal immune cells (*n* = 92, 73%)	Tumor with intermediate/heterogeneous infiltrate (*n* = 25, 20%)	Tumor with high immune infiltrate (*n* = 9, 7%)	
Age, years				
Median (range)	52 (29–82)	56 (38–75)	61 (47–77)	
Mean ± SD	52.8 ± 11.6	55.8 ± 11.1	61.2 ± 8.7	
T				
T1	37 (71)	10 (19)	5 (10)	0.821
T2	47 (65)	15 (23)	3 (5)	0.744
T3	8 (89)	0	1 (11)	0.307
N				
N0	44 (69)	16 (25)	4 (6)	0.726
N1	32 (74)	8 (19)	3 (7)	1
N2	9 (82)	1 (9)	1 (9)	0.748
N3	6 (86)	0	1 (14)	0.290
Nx^b^	1 (100)	0	0	
M				
M0	79 (75)	20 (19)	6 (6)	0.919
Mx^c^	13 (62)	5 (24)	3 (14)	0.361
Stage				
I	22 (73)	6 (20)	2 (7)	1
II	53 (70)	18 (24)	5 (7)	0.826
III	17 (85)	1 (5)	2 (10)	0.204
Histological type				
Invasive ductal carcinoma	80 (75)	19 (18)	7 (7)	0.921
Invasive lobular carcinoma	10 (59)	5 (29)	2 (12)	0.371
Other	2 (67)	1 (33)	0	
Estrogen receptor status				
Positive	79 (75)	19 (18)	7 (7)	0.947
Negative	13 (62)	6 (29)	2 (10)	0.560
Progesterone receptor status				
Positive	71 (76)	16 (17)	6 (6)	0.888
Negative	21 (64)	9 (27)	3 (9)	0.515
Human epidermal growth factor receptor 2 status				
Positive	14 (61)	6 (26)	3 (13)	0.384
Negative	76 (76)	19 (19)	5 (5)	0.790
Equivocal	2 (67)	0	1 (33)	
IHC subtype				
HR+/HER2−	68 (78)	14 (16)	5 (6)	0.824
HER2+	14 (61)	6 (26)	3 (13)	0.571
ER−/PR−/HER2−	10 (63)	5 (31)	1 (6)	0.592
PAM50 intrinsic subtype				
Luminal A	53 (75)	13 (18)	5 (7)	0.966
Luminal B	22 (79)	4 (14)	2 (7)	0.875
HER2	4 (50)	3 (38)	1 (12)	0.219
Basal	10 (63)	5 (31)	1 (6)	0.536
Normal	3 (100)	0	0	

*Abbreviations: ER* Estrogen receptor, *HER2* Human epidermal growth factor receptor 2, *HR* Hormone receptor, *PR* Progesterone receptor, *TIL* Tumor-infiltrating lymphocyte

^a^Fisher’s exact test was used to compare TIL distribution within selected category with TIL distribution of whole population

^b^Lymph node stage is not available

^c^Metastasis cannot be measured

### Imaging features associated with tumor-infiltrating lymphocytes

**Fig. 2:**
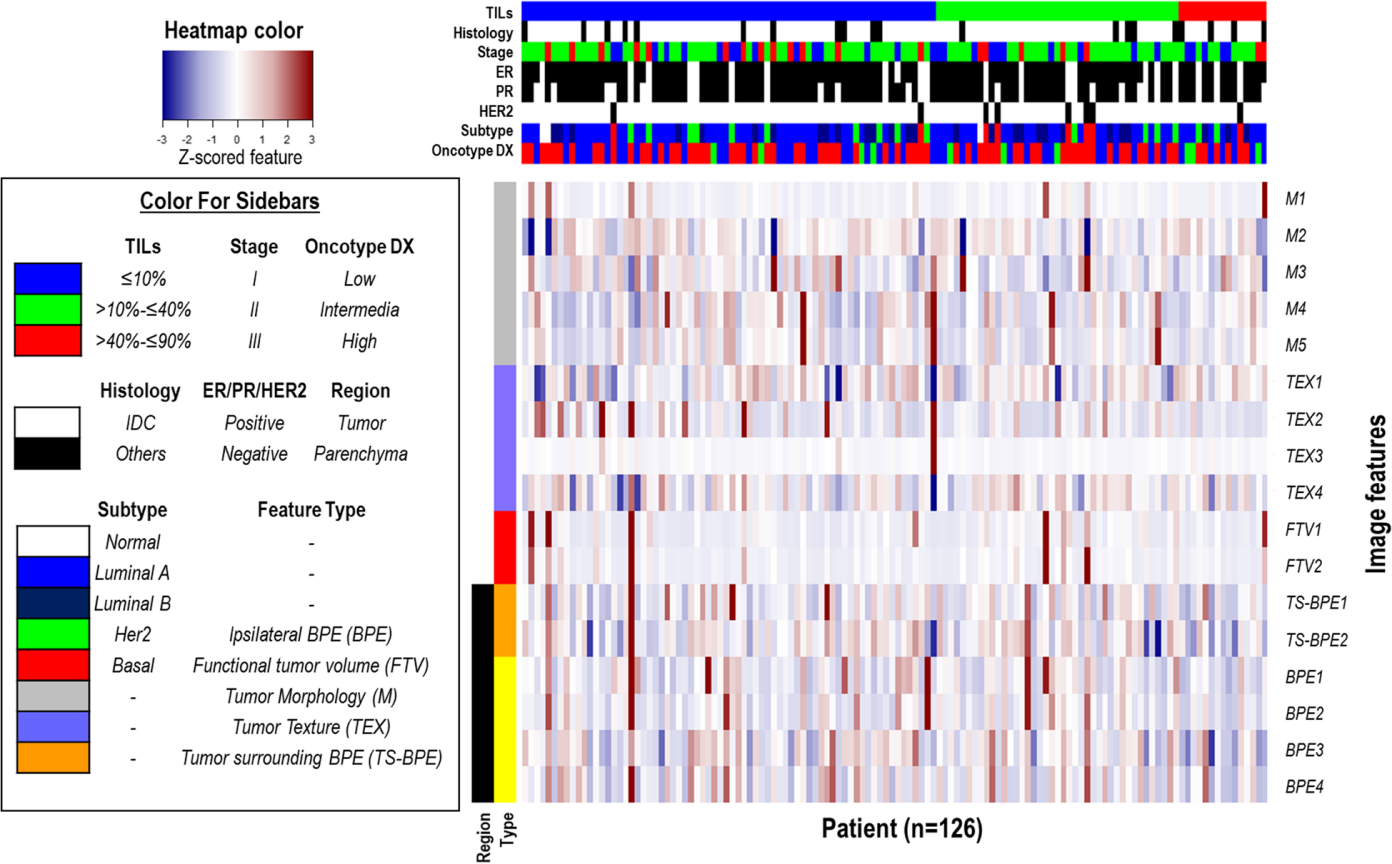
Heat map of computational imaging features from The Cancer Genome Atlas cohort. In the plot, all 17 features (presented in each row and color-coded by the region and type) from 126 patients (presented in each column) were ranked by their TILs (monotonically increasing from left to right). All imaging features were standardized to have a zero mean and unit standard deviation. Imaging features were defined in Table 1. *BPE* Background parenchymal enhancement, *ER* Estrogen receptor, *HER2* Human epidermal growth factor receptor 2, *IDC* Invasive ductal carcinoma, *PR* Progesterone receptor, *TIL* Tumor-infiltrating lymphocyte

**Fig. 3:**
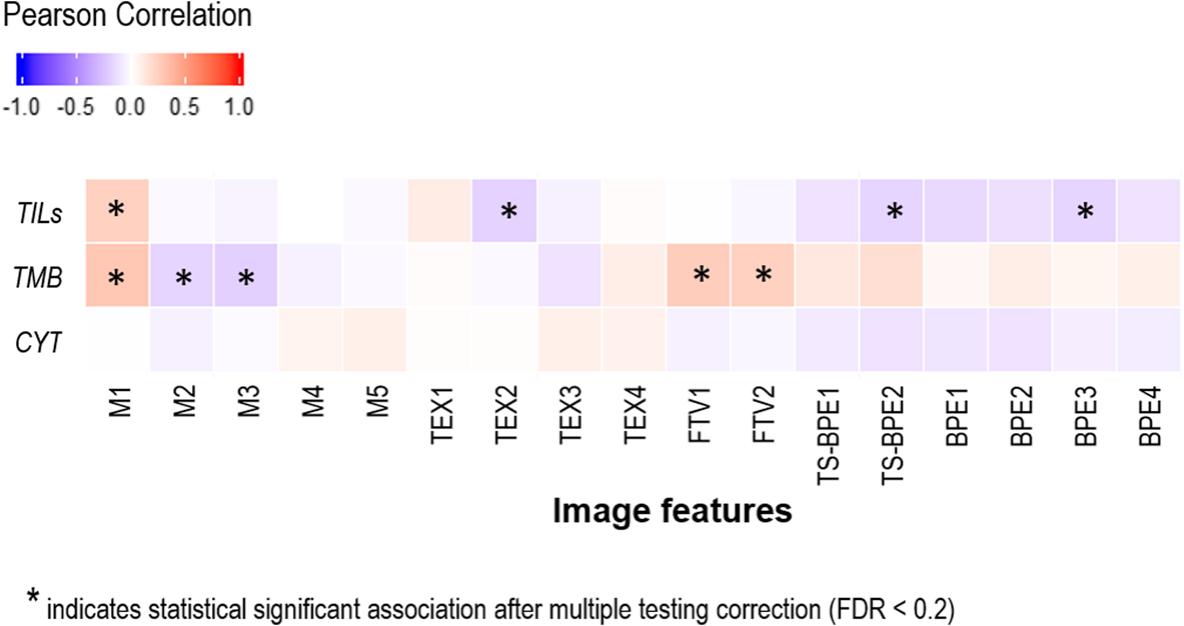
Heat map of correlation between 17 imaging features and tumor-infiltrating lymphocytes (TILs) from pathologists’ reading, nonsynonymous tumor mutation burden (TMB), and cytolytic activity (CYT). *FDR* False discovery rate

**Fig. 4:**
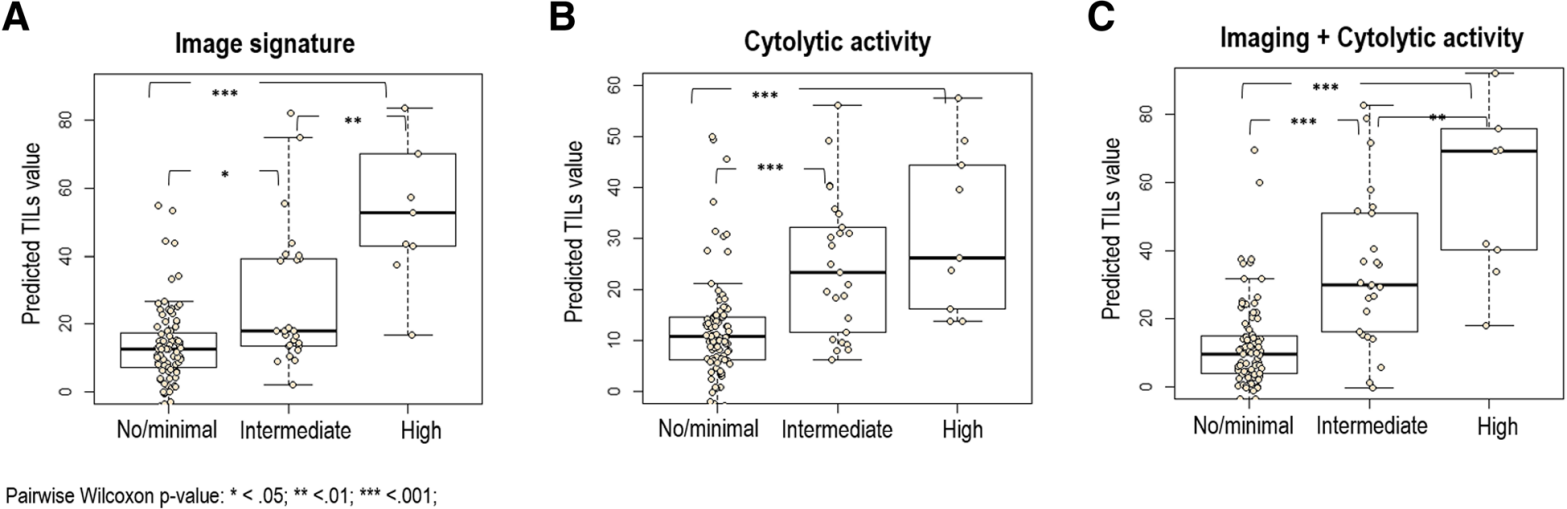
Box plots of the predicted tumor-infiltrating lymphocyte (TIL) values stratified by the original pathologists’ reading in The Cancer Genome Atlas cohort through (**a**) the imaging signature, (**b**) cytolytic activity score, and (**c**) the composite model

**Fig. 5:**
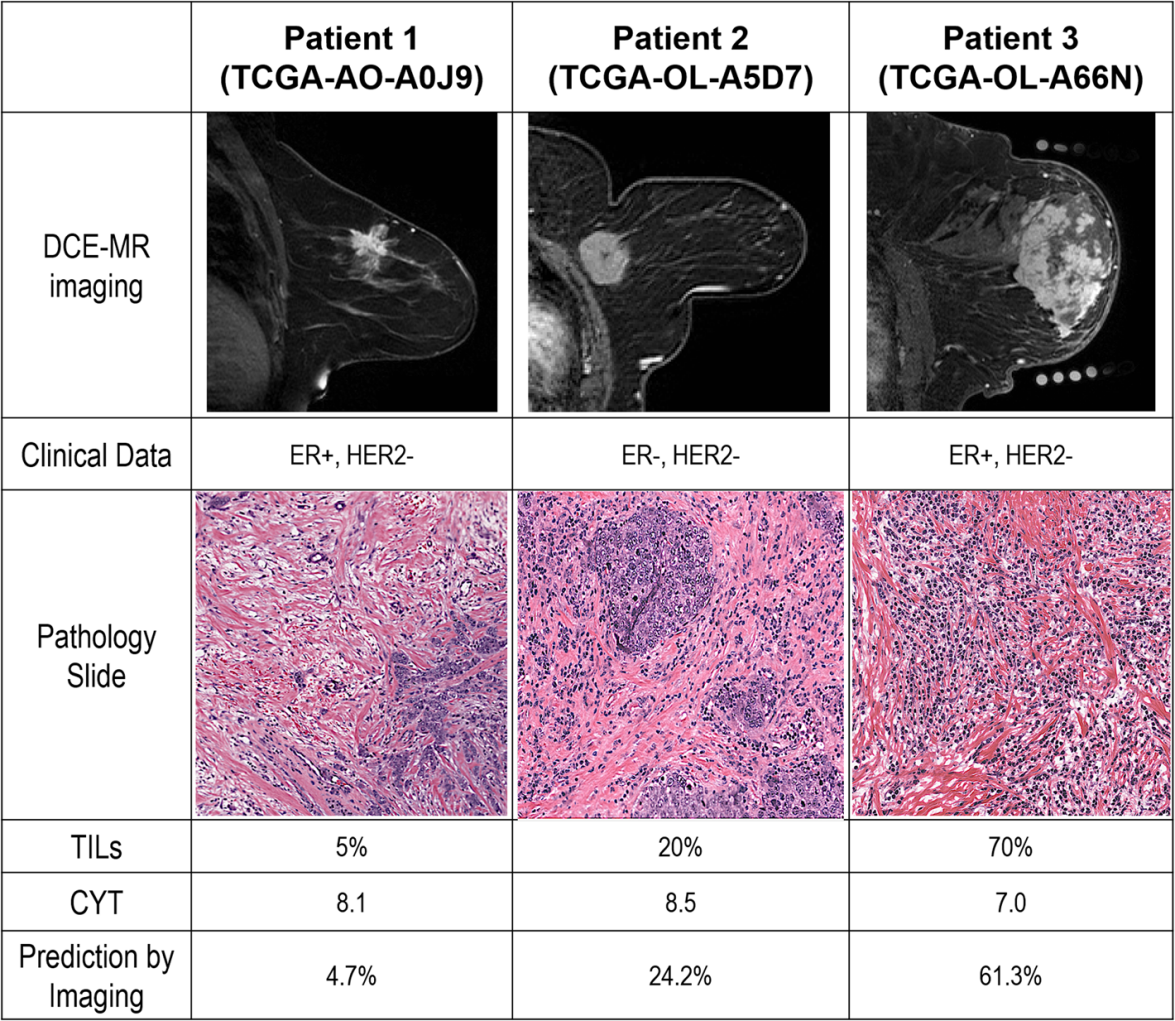
Illustration of three patients with breast cancer, where the proposed magnetic resonance (MR) imaging signature accurately predicts their tumor-infiltrating lymphocytes (TILs) from pathologists’ reading. *CYT* Cytolytic activity, *ER* Estrogen receptor, *HER2* Human epidermal growth factor receptor 2, *DCE* Dynamic contrast-enhanced, *TCGA* The Cancer Genome Atlas

Each of the 17 imaging features independently characterizes the cancer phenotypes, and their pairwise correlation map is shown in Additional file 1: Figure S2. Figure 2 shows the heat map of 17 imaging features for 126 patients in TCGA cohort ranked on the basis of their TILs, monotonically increasing from left to right. In the univariate analysis, 4 of 17 imaging features were significantly associated with the percentage of TILs (*P* < 0.05 and FDR < 0.2), as shown in Fig. 3. Among these four features, the tumor volume was positively correlated with TILs, whereas cluster shade of signal enhancement ratio (SER) map, mean SER of tumor surrounding BPE, and proportion of BPE were negatively correlated with TILs (Additional file 2: Table S2). Next, we built an imaging signature for TILs by fitting a linear model, which consisted of five imaging features: 4.4 × *M*1 − 3.14 × *TEX*2 − 2.0 × *TS* − *BPE*2 − 2.62 × *BPE*1 − 0.72 × *BPE*3 + 13.02, where *M1* = tumor volume, *TEX2* = cluster shade of SER map, *TS-BPE2* = mean SER of tumor surrounding BPE (2 cm), *BPE1* = BPE volume (percentage enhancement or PE > 20%), and *BPE3* = BPE proportion (PE, > 20%). The mean and SD values of the five selected imaging features are shown in Additional file 2: Table S3. This imaging signature had a moderate linear correlation with TILs (*ρ* = 0.40; 95% CI, 0.24–0.54; *P* = 4.2E-6). Moreover, the imaging signature is able to separate three TILs categories in pairwise fashion (Fig. 4a), with prediction accuracy of 0.73, 0.71, and 0.71, respectively (Table 3). Figure 5 showed the details of three representative patients where there is good agreement between the predicted TILs from proposed imaging signatures and TIL readings by two pathologists.

**Table 3:** Model evaluation of three classification models for predicting tumor-infiltrating lymphocyte groups in The Cancer Genome Atlas

	Specificity	Sensitivity	Accuracy
Low vs intermediate TIL groups			
Imaging signature	0.93	0.36	0.73
Cytolytic activity	0.86	0.68	0.83
Imaging + cytolytic activity	0.82	0.68	0.85
Low vs high TIL groups			
Imaging signature	0.70	0.89	0.71
Cytolytic activity	0.68	1	0.71
Imaging + cytolytic activity	0.91	0.88	0.91
Intermediate vs high TIL groups			
Imaging signature	0.64	0.89	0.71
Cytolytic activity	0.84	0.44	0.74
Imaging + cytolytic activity	0.68	0.78	0.75

*TIL* Tumor-infiltrating lymphocyte

### Relationships between imaging, molecular signatures, and tumor-infiltrating lymphocytes

We evaluated the associations between imaging and immune-related molecular features, as well as the percentage of TILs, in TCGA cohort. In addition to the imaging signature, cytolytic score was significantly associated with TILs (*ρ* = 0.51; 95% CI, 0.36–0.63; *P* = 1.6E-9) (Fig. 4b), whereas none of the clinicopathological factors or the somatic mutation burden were correlated with TILs (Table 4). We found that five imaging features were significantly associated with mutation burden, but none was associated with cytolytic score (Fig. 3, Additional file 2: Table S4). This suggests that imaging and cytolytic score are independent and could be complementary to each other for predicting TILs. For three cases in Fig. 5, the imaging signature can provide more accurate prediction of TILs than the model of cytolytic score.

**Table 4:** Univariate and multivariate analyses of tumor-infiltrating lymphocytes using the imaging signature, clinicopathological factors, and molecular features in The Cancer Genome Atlas Cohort

Predictors	Univariate			Multivariate		
	ρ	95% CI	*P* value	Coefficient	SE	*P* value
Imaging signature	0.40	0.24–0.54	< 0.0001^a^	4.78	1.60	0.003^a^
T^b^	–	–	0.269	–	–	–
N^b^	–	–	0.799	–	–	–
M^b^	–	–	0.214	–	–	–
Stage^b^	–	–	0.650	−2.85	1.97	0.151
ER^c^	–	–	0.479	−3.42	6.85	0.618
PR^c^	–	–	0.561	2.71	4.05	0.504
HER2^b^	–	–	0.152	3.51	3.42	0.308
Triple-negative^c^	–	–	0.782	−0.51	7.43	0.945
PAM50 subtype^b^	–	–	0.309	–	–	–
Mutation burden	0.13	−0.05-0.30	0.167	−0.20	1.24	0.870
Cytolytic activity	0.51	0.36–0.63	< 0.0001*	7.69	1.27	< 0.000^a^

*Abbreviations: ER* Estrogen receptor, *HER2* Human epidermal growth factor receptor 2, *PR* Progesterone receptor

^a^*P* < 0.05

^b^For multinomial variables, the Kruskal-Wallis test was used

^c^For binary variables, the *t* test was used

### Composite model for tumor-infiltrating lymphocytes

On multivariate analysis, the imaging signature and cytolytic score remained as independent predictors of TILs (*P* = 0.004 and *P* < 0.0001, respectively) after adjusting for stage, estrogen receptor/progesterone receptor/HER2 status, and mutation burden (Table 4). We retained both significant variables and refitted a composite model for predicting TILs: 5.86 × *Imaging Signature* + 7.78 × *Cytolytic Score* + 13.0. The linear correlation between the composite model and TILs was improved (*ρ* = 0.62; 95% CI, 0.50–0.72; *P* = 9.7E-15). Detailed box plots of inferred TILs from the composite model vs the original pathologists’ readings are presented in Fig. 4c.

**Fig. 6:**
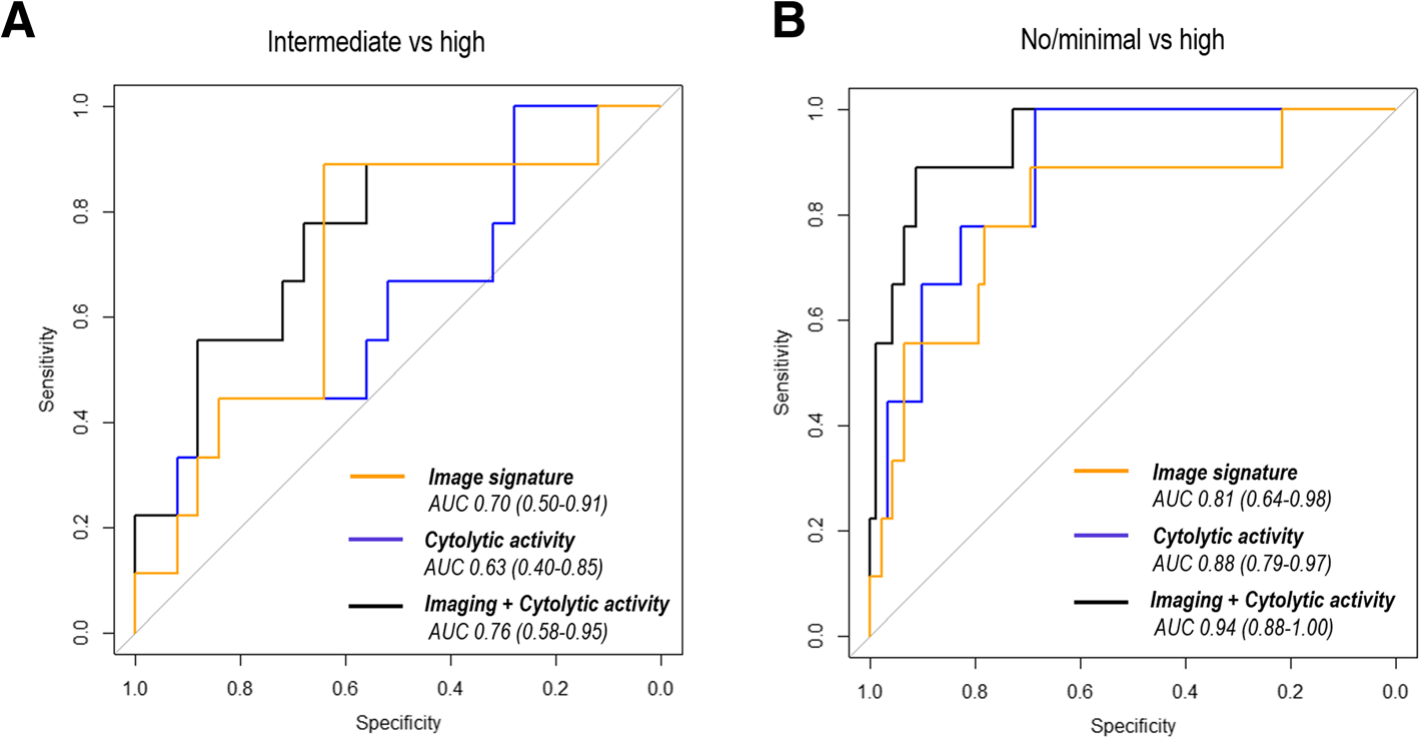
ROC curves constructed by using imaging signature, cytolytic activity, and the composite model for classification of (**a**) intermediate vs high tumor-infiltrating lymphocyte (TIL) groups and (**b**) no/minimal vs high TIL groups

We tested the composite model for predicting three predefined TILs categories. As shown in Fig. 6a, cytolytic score alone could not differentiate between intermediate and high TILs (AUC, 0.63; *P* = 0.14). By integrating imaging signature and cytolytic score, the composite model successfully separated these two TILs groups (AUC, 0.76; *P* = 0.01), and the improvement was statistically significant (DeLong test *P* = 0.039). Similar results were observed for differentiating low vs high TILs groups (AUC, 0.88 vs 0.94) (Fig. 6b). For distinguishing low and intermediate groups, there was no significant improvement using the composite model over cytolytic score (AUC, 0.77 vs 0.79) (Additional file 1: Figure S3). In addition, we performed a detailed evaluation of the proposed composite model, as in Table 3, where the composite model’s accuracy is 0.85, 0.91, and 0.75, respectively.

### Clinical validation of the composite model

**Fig. 7:**
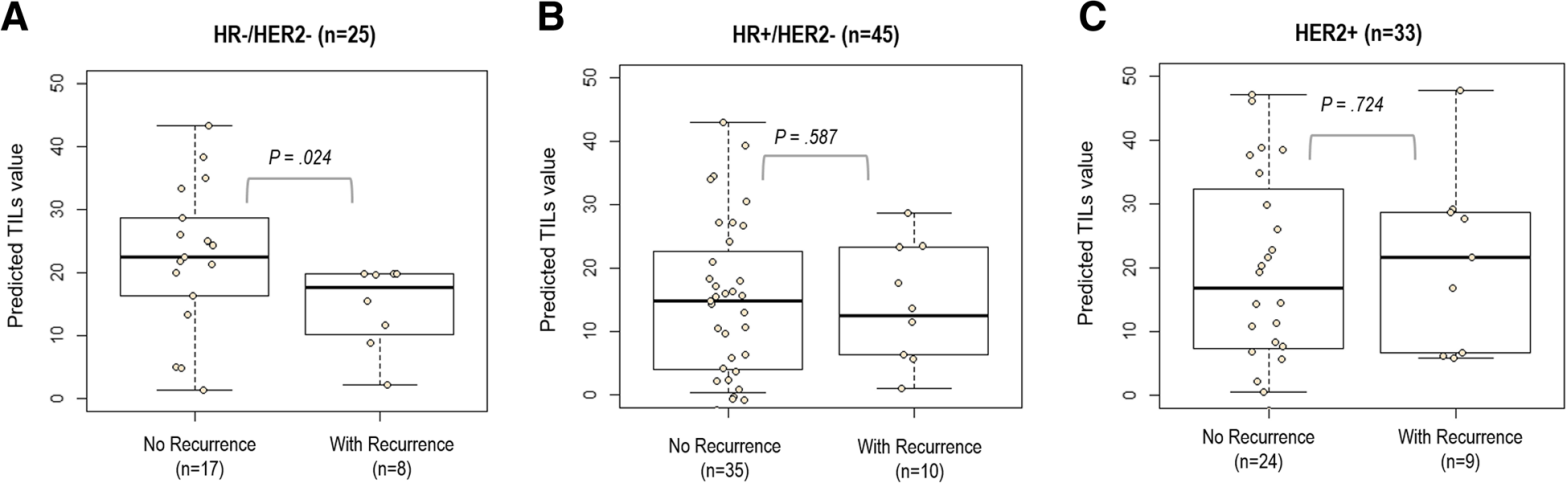
Predicted tumor-infiltrating lymphocyte (TIL) values for I-SPY patients based on the proposed composite model, stratified by recurrence status in box plots for (**a**) hormone receptor-negative (HR−)/human epidermal growth factor receptor 2-negative (HER2−), (**b**) HR+/HER2−, and (**c**) HER2+ patients

**Fig. 8:**
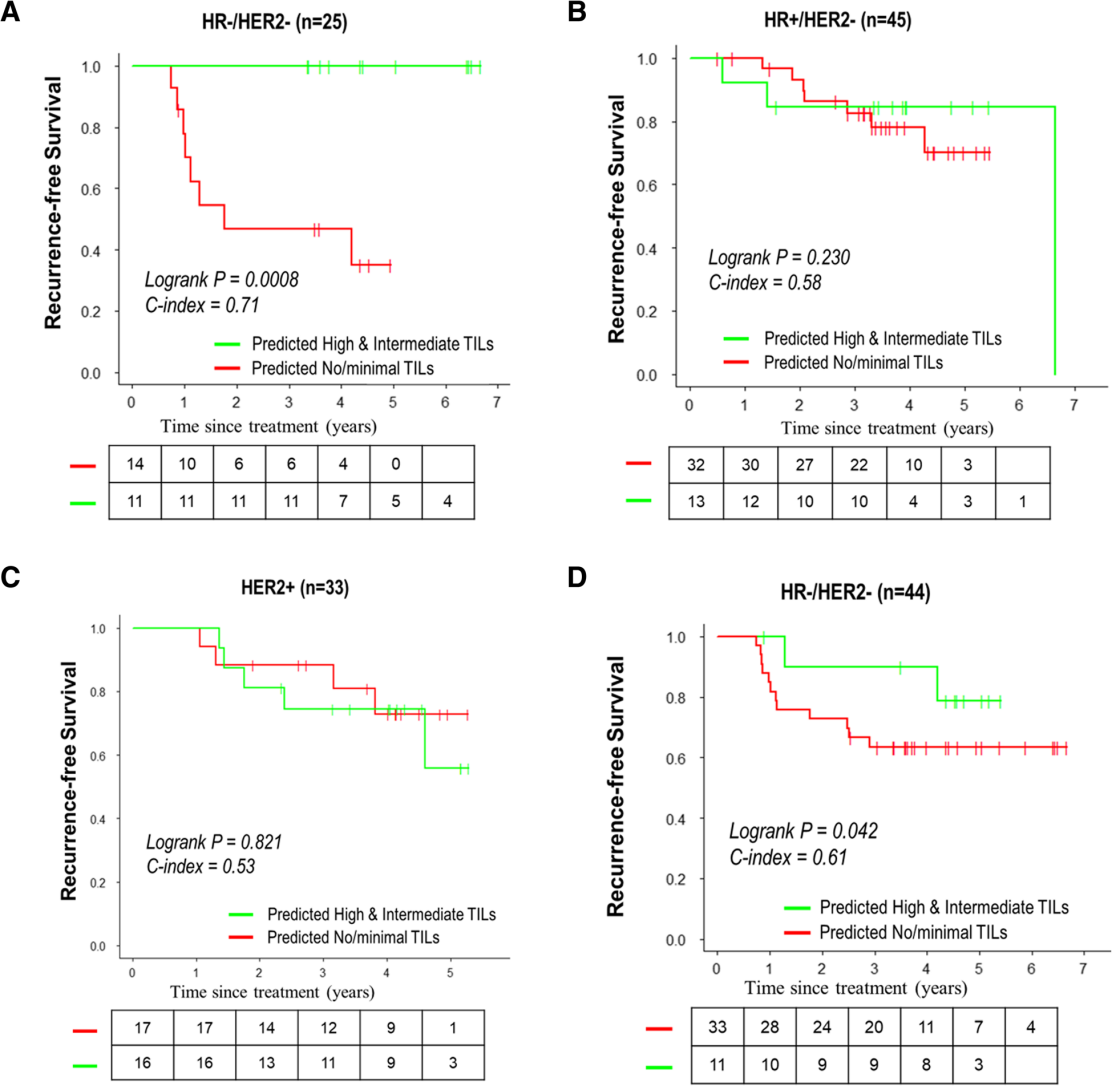
Kaplan-Meier curve of recurrence-free survival for I-SPY patients, stratified by predicted tumor-infiltrating lymphocyte (TIL) groups (no/minimal vs high/intermediate). **a** Composite model for hormone receptor-negative (HR−)/human epidermal growth factor receptor 2-negative (HER2−) breast cancer. **b** Composite model for HR+/HER2− breast cancer. **c** Composite model for HER2+ breast cancer. **d** Imaging signature for HR−/HER2− breast cancer

The previously developed composite model was used to infer TILs based on imaging and molecular data in an independent cohort from the I-SPY 1 trial. We found that hormone receptor-negative (HR−)/HER2− or TNBC had significantly higher predicted TILs than HR+/HER2− breast cancer (*P* = 0.049) (Additional file 1: Figure S4). With the threshold values obtained from the training cohort, we divided the patients into three groups based on the predicted TIL values. Then, we investigated the relationship between predicted TIL groups and outcomes. In TNBC, patients without recurrence had significantly higher predicted TILs than those who developed recurrence (*P* = 0.024) (Fig. 7a). Within the TNBC group, distinct RFS exists between the predicted no/minimal TIL group and the predicted high/intermediate TILs group (log-rank *P* = 0.0008) (Fig. 8a), where the group with lower TILs had significantly worse prognosis. However, predicted TIL groups were not associated with RFS in HR+/HER2− or HER2+ breast cancer (Fig. 7b and c and Fig. 8b and c, respectively).

Additionally, to validate the imaging signature for TILs, we applied it to 44 patients with TNBC who had images publicly available in the I-SPY 1 cohort. Similarly, with the threshold value obtained from the training cohort, we classified the patients into three TIL categories. A trend similar to that for the composite mode was observed, where no/minimal TILs had a significantly worse prognosis than high/intermediate TILs regarding their RFS (log-rank *P* = 0.042) (Fig. 8d).

## Discussion

In this study, we aimed to dissect the complex tumor-immune interactions in breast cancer [5, 6] by integrating imaging, genomic, and histological data. In particular, our pilot study showed that the percentage of stromal TILs evaluated on histological tissue sections were significantly associated with specific enhancement patterns of tumor and surrounding parenchyma at DCE-MRI. Our findings are consistent with a recent study that demonstrated a link between heterogeneous enhancement of tumor-adjacent parenchyma on DCE-MRI and dysregulated tumor necrosis factor signaling pathway in breast cancer [47]. Both studies support the role of inflammatory or immune response in breast cancer progression and its relationship to specific parenchymal enhancement patterns at DCE-MRI. Consistent with previous work, we found that TILs were also associated with cytolytic activity but not with tumor mutational burdens in TCGA breast cancer cohort [48]. In addition to identifying associations, we further developed and evaluated prediction models for TILs that integrated imaging and genomic data. Our results show that a composite model combining an imaging signature and cytolytic score achieved augmented linear correlation with TILs compared with using either alone. The composite model showed good or excellent discriminative ability among low, intermediate, and high TIL groups, with AUCs ranging from 0.76 to 0.94.

The reliable evaluation of TILs has significance and clinical implications in breast cancer. Abundant evidence has demonstrated that TILs have strong prognostic and predictive value in specific breast cancer subtypes [6]. For localized breast cancer, TILs have been shown to be associated with pathological complete response and prognosis after chemotherapy or targeted therapies for localized breast cancer. This suggests that our imaging and molecular signature for TILs could help select patients who will be most likely respond to and benefit from neoadjuvant chemotherapy or targeted therapies in locally advanced breast cancer. On the other hand, they may also be used in combination with established clinical and pathological criteria to identify low-risk patients with a favorable prognosis who might be spared adjuvant chemotherapy in early-stage breast cancer. However, this will require further validation in prospective clinical studies. Although there are yet no U.S. Food and Drug Administration-approved immunotherapies for breast cancer, numerous trials are ongoing with the goal of assessing the clinical activity and potential benefit of ICB [49]. Because the success of ICB hinges on a preexisting antitumor immunity, which is manifested as TILs, TILs could serve as useful predictive biomarkers to select patients who are likely to benefit from immunotherapy.

The current gold standard for evaluating TILs is based on pathologists’ visual assessment of H&E-stained whole-tumor tissue sections. This approach is limited mainly by intra- and interrater variability [16]. Gene expression profiles can also reflect antitumor immune response. Recently, a two-gene cytolytic score was proposed to characterize local immune infiltration and cytolytic activity in a large study across 18 tumor types in TCGA [40]. Nonetheless, pathological or molecular evaluation of TILs may be confounded by the spatial intratumoral heterogeneity, especially in the neoadjuvant setting with core biopsies [50]. On the other hand, imaging provides a global, unbiased picture of the entire tumor and its surrounding tissue, potentially allowing more reproducible evaluation for TILs. Our results demonstrate that, compared with cytolytic score, the imaging signature may be particularly useful in distinguishing tumors with high vs intermediate TILs. Although imaging analysis alone cannot replace pathological evaluation for TILs, our study supports imaging playing an important role in this process by providing key complementary information in equivocal cases or situations that are prone to sampling bias (e.g., in core biopsy). In our study, given the tumor contours manually delineated by radiologists, the subsequent imaging analysis was fully automatic. With the rapid advancement of machine learning in radiology [51], we anticipate that much of the process will be automated and that radiological interpretation bias can be minimized. One unique advantage of MRI is that it provides a global view of the whole tumor as well as its surrounding parenchyma, which overcomes the issue of sampling bias in core biopsy.

We validated the clinical relevance of our composite prediction model for TILs in an independent cohort. Consistent with previous findings [52], we showed that TNBC had significantly higher predicted TILs than HR+/HER2− breast cancer. So far, the strongest evidence for the prognostic value of TILs has been in TNBC, whereas its significance is more mixed in HER2+ and seems uncertain in HR+/HER2− subtypes [6]. We confirmed that higher TILs predicted by the composite model were indeed associated with better prognosis and RFS in TNBC, but not among other subtypes. Given the relatively small number of TNBC cases in the I-SPY 1 cohort, it would be important to further validate the model in future studies with more patients.

Our study adds to the growing body of literature where a more detailed comprehensive analysis of imaging phenotypes could reveal the underlying tumor pathophysiology at the molecular or pathological level [17–28, 53–55]. Different from previous radiogenomic studies that focused on analyses of imaging and genomic properties of the tumor, our study focused on immune infiltration in the stroma and included imaging features of the tumor as well as its surrounding parenchymal tissue. Another distinction is that previous work aimed to find correlation (i.e., similarity) between imaging and molecular data, whereas our study demonstrates that imaging can provide independent value and complement molecular profiles for predicting TILs.

There are several limitations of this study. The images and samples in TCGA cohort were retrospectively collected, which may not be a representative patient population for breast cancer. The association findings in this study should be interpreted as hypothesis-generating, and the composite prediction model for TILs requires validation in large, ideally prospective cohorts. Owing to the limited sample size, our analysis may have been insufficiently powered to detect differences in TILs by receptor status. Future work is needed to confirm the findings in a subtype-specific manner. In addition, there are diverse imaging acquisition protocols in the multi-institutional TCGA cohort, which may have confounded our analysis. Despite our efforts to harmonize imaging data, uncertainty could remain. Finally, we focused on DCE-MRI for association with TILs. Additional imaging modalities such as T2-weighted and diffusion-weighted MRI may be incorporated in future studies.

## Conclusions

We showed that specific tumoral and parenchymal imaging features are associated with TILs and that integration of imaging and molecular features allows for better prediction of TILs in breast cancer. These preliminary findings should be validated in additional larger studies.

## Additional files

Additional file 1: **Figure S1.** Flowcharts of detailed patient selection for both TCGA and I-SPY 1 trials in the proposed study. **Figure S2.** Pairwise Pearson’s correlation of 17 quantitative DCE-MRI features (*see definition* in Table 1). **Figure S3.** ROC curves corresponding to mutation burden, cytolytic activity, and proposed composite model for classification of low vs intermediate TIL groups. **Figure S4.** Predicted TIL values for I-SPY patients based on the composite model, stratified by (**a**) three subtypes and (**b**) recurrence status. (DOCX 600 kb)

Additional file 2: **Table S1.** Clinical and pathological characteristics for eligible patients in the I-SPY cohort. **Table S2.** Imaging features associated with tumor-infiltrating lymphocytes (TILs) with FDR < 0.2. **Table S3.** Mean and SD values for five quantitative imaging features. **Table S4.** Imaging features associated with nonsynonymous mutation burdens with FDR < 0.2. (DOCX 17 kb)
