# Supplementary material for: Magnetic resonance imaging and molecular features associated with tumor-infiltrating lymphocytes in breast cancer
Source: Breast Cancer Res. 2018 Sep 3;20:101. doi: 10.1186/s13058-018-1039-2 (PMC6122724; doi:10.1186/s13058-018-1039-2)
Supplement: Supplementary file 1 — Figure S1. Flowcharts of detailed patient selection for both TCGA and I-SPY 1 trials in the proposed study. Figure S2. Pairwise Pearson’s correlation of 17 quantitative DCE-MRI features (see definition in Table 1). Figure S3. ROC curves corresponding to mutation burden, cytolytic activity, and proposed composite model for classification of low vs intermediate TIL groups. Figure S4. Predicted TIL values for I-SPY patients based on the composite model, stratified by (a) three subtypes and (b) recurrence status. (DOCX 600 kb) [file 13058_2018_1039_MOESM1_ESM.docx]

**
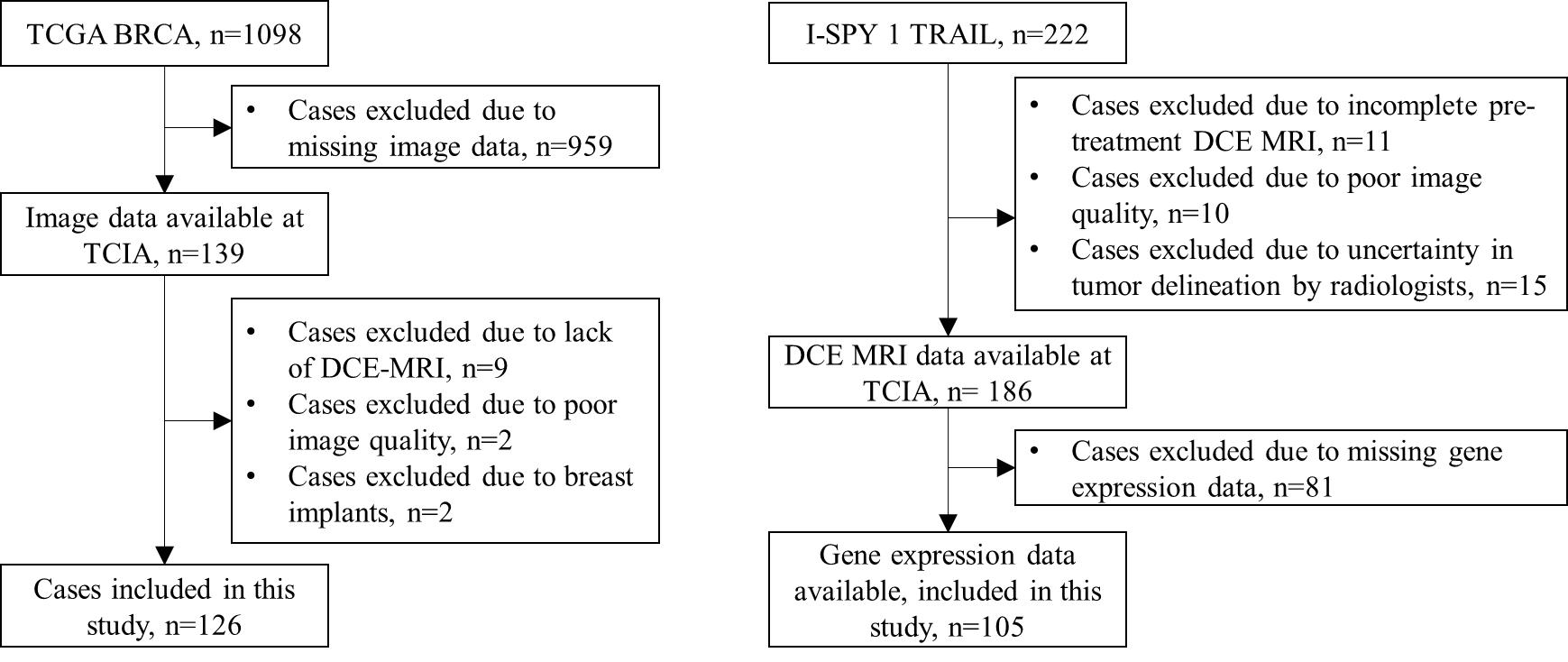
**

**Figure S1.** Flowcharts of detailed patient’s selection for both TCGA and I-SPY 1 trail in the proposed study


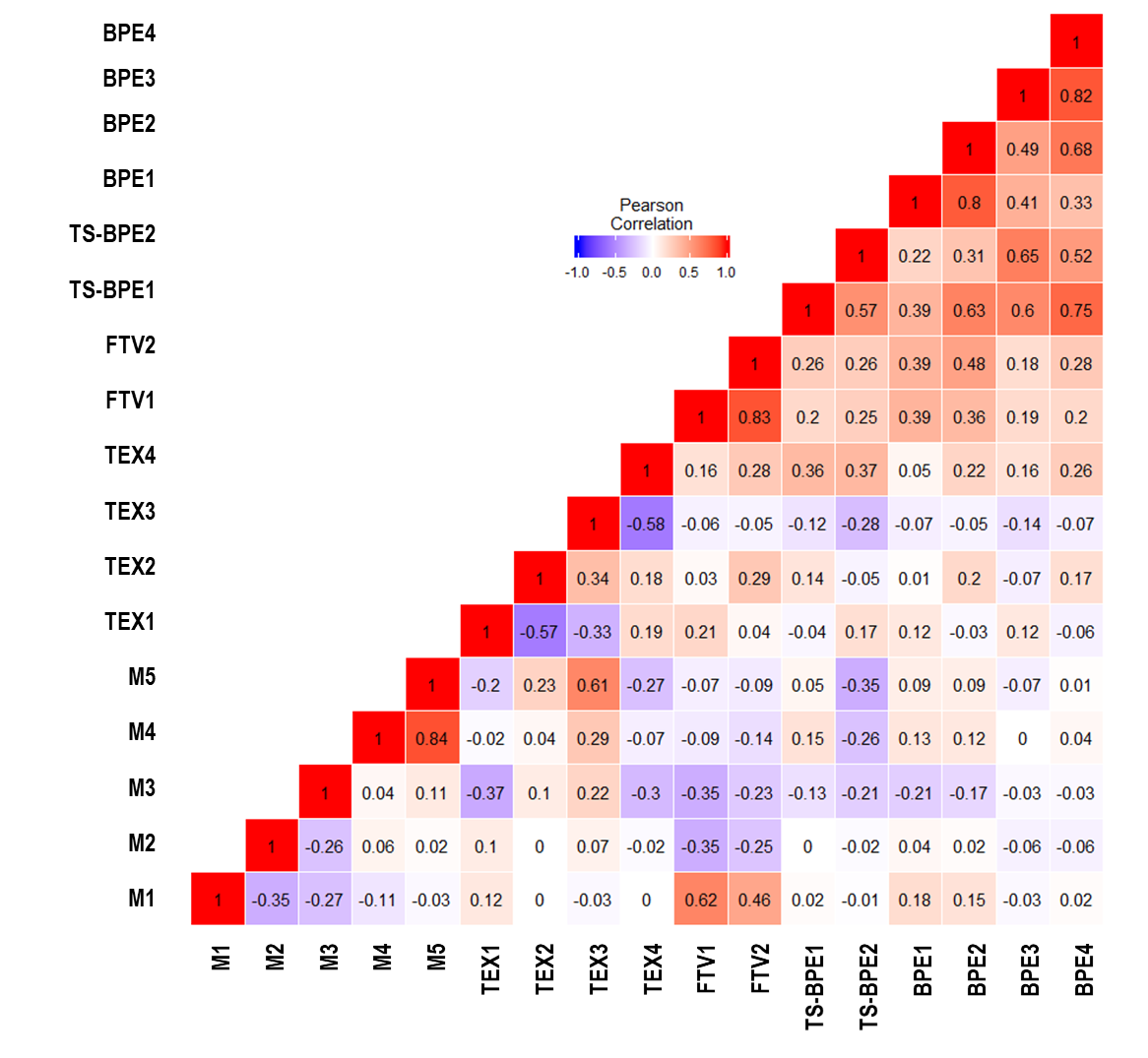


**Figure S2.** Pair-wise Pearson’s correlation of 17 quantitative DCE-MR imaging features (see definition in Table 1)

**
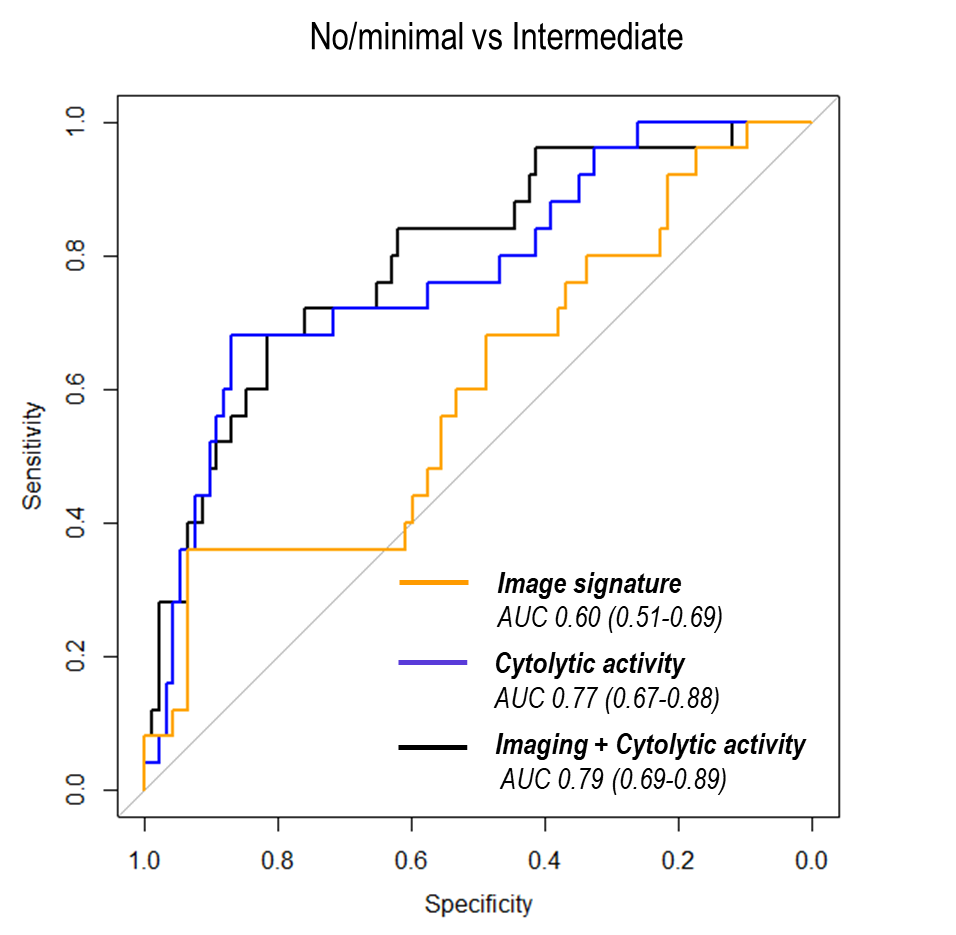
**

**Figure S3.** ROC curves corresponding to mutation burden, cytolytic activity, and proposed composite model, for classification of low versus intermediate TILs groups

**
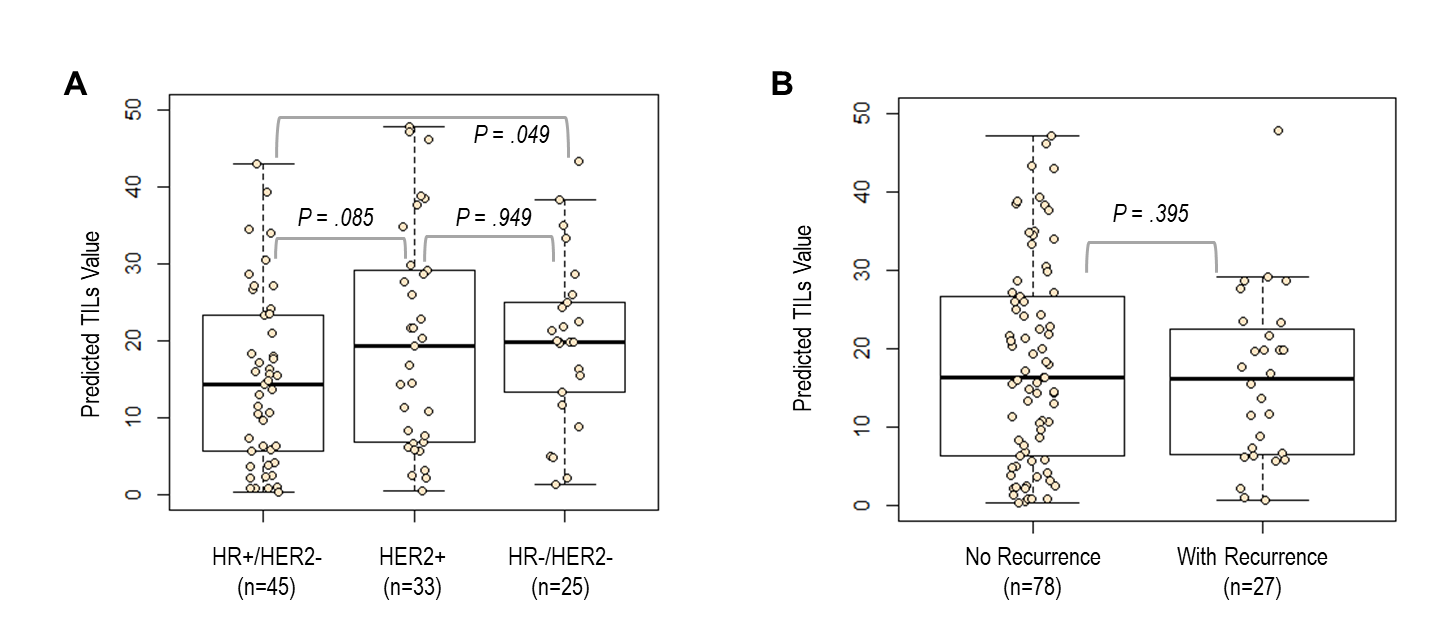
**

**Figure S4.** Predicted TILs values for I-SPY patients based on the composite model, stratified by A) three subtypes and B) recurrence status
