# Supplementary material for: Magnetic resonance imaging and molecular features associated with tumor-infiltrating lymphocytes in breast cancer
Source: Breast Cancer Res. 2018 Sep 3;20:101. doi: 10.1186/s13058-018-1039-2 (PMC6122724; doi:10.1186/s13058-018-1039-2)
Supplement: Supplementary file 2 — Table S1. Clinical and pathological characteristics for eligible patients in the I-SPY cohort. Table S2. Imaging features associated with tumor-infiltrating lymphocytes (TILs) with FDR < 0.2. Table S3. Mean and SD values for five quantitative imaging features. Table S4. Imaging features associated with nonsynonymous mutation burdens with FDR < 0.2. (DOCX 17 kb) [file 13058_2018_1039_MOESM2_ESM.docx]

**Table S1.** Clinical and pathologic characteristics for eligible patients in the I-SPY cohort

|  | **I-SPY (n=105)** | **TCGA (n=126)** | **P-value** |
| --- | --- | --- | --- |
| Age (y) |  |  | 0.008 |
| Median (range) | 48.1 (28.8-65.4) | 53 (29-82) |  |
| Mean ± standard deviation | 47.7 ± 8.4 | 54.0 ± 11.5 |  |
| Stage^†^ |  |  | <0.001 |
| I | 1% | 24% |  |
| II | 47% | 60% |  |
| III | 43% | 16% |  |
| Estrogen receptor status |  |  | <0.001 |
| Positive | 57 | 105 |  |
| Negative | 48 | 21 |  |
| Progesterone receptor status |  |  | <0.001 |
| Positive | 46 | 93 |  |
| Negative | 59 | 33 |  |
| Human epidermal growth factor receptor 2 status |  |  | <0.001 |
| Positive | 33 | 23 |  |
| Negative | 70 | 100 |  |
| Equivocal | 0 | 3 |  |
| Unknown | 2 | 0 |  |
| IHC subtype |  |  | <0.001 |
| HR+/HER2- | 45 | 87 |  |
| HER2+ | 33 | 23 |  |
| TN | 25 | 16 |  |
| Unknown | 2 | 0 |  |
| Follow-up (years) |  |  |  |
| Median (range) | 4.0 (0.5-6.7) | - |  |
| Mean ± standard deviation | 4.0 ± 1.2 | - |  |
| Recurrence |  |  |  |
| Yes | 28 | - |  |
| No | 77 | - |  |
| **Note:** ^†^ the stage distribution for I-SPY cohort was extracted from the whole I-SPY 1 Trial (n=221). | | | |

**Table S2.** Image features associated with tumor-infiltrating lymphocytes (TILs), with FDR < 0.2.

|  | **ρ** | **95% CI** | **P-value** | **FDR** |
| --- | --- | --- | --- | --- |
| Volume | 0.24 | 0.07-0.40 | 0.006 | 0.10 |
| Cluster Shade of SER Map | -0.19 | -0.35 - -0.01 | 0.038 | 0.17 |
| Mean SER of Tumor Surrounding BPE (2cm) | -0.18 | -0.35 - -0.01 | 0.042 | 0.17 |
| BPE proportion (PE>20%) | -0.18 | -0.35 - -0.01 | 0.038 | 0.17 |

**Table S3.** Mean and standard deviation values for 5 quantitative imaging features

| **Image Features** | **TCGA (n=126)** | **I-SPY (n=105)** | **P-value** |
| --- | --- | --- | --- |
| M1: Volume | 10.4 (30.0) | 22.1 (49.9) | 0.018 |
| TEX2: Cluster Shade of SER Map | 2525.9 (4441.2) | 2714.2 (4961.2) | 0.382 |
| TS-BPE2: Mean SER of Tumor Surrounding BPE (2cm) | 0.5 (0.2) | 0.6 (0.2) | <0.001 |
| BPE1: BPE volume (PE>20%) | 121.7 (118.2) | 185.7 (182.9) | 0.004 |
| BPE3: BPE proportion (PE>20%) | 0.5 (0.2) | 0.5 (0.2) | 1 |

**Table S4.** Image features associated with non-synonymous mutation burdens, with FDR < 0.2

|  | **ρ** | **95% CI** | **P-value** | **FDR** |
| --- | --- | --- | --- | --- |
| Volume | 0.29 | 0.12-0.44 | 0.001 | 0.02 |
| Sphericity | -0.18 | -0.34 - -0.01 | 0.047 | 0.16 |
| Surface to volume ratio | -0.20 | -0.36 - -0.02 | 0.030 | 0.12 |
| FTV (SER>1.0) | 0.26 | 0.09-0.42 | 0.003 | 0.02 |
| FTV (SER>1.5) | 0.24 | 0.07-0.40 | 0.008 | 0.04 |
